# Assessment of intramyocardial hemorrhage by T1-weighted cardiovascular magnetic resonance in reperfused acute myocardial infarction

**DOI:** 10.1186/1532-429X-14-59

**Published:** 2012-08-30

**Authors:** Steen Fjord Pedersen, Samuel A Thrysøe, Michael P Robich, William P Paaske, Steffen Ringgaard, Hans Erik Bøtker, Esben S S Hansen, Won Yong Kim

**Affiliations:** 1 Department of Cardiothoracic and Vascular Surgery T, Aarhus University Hospital Skejby, Brendstrupsgaardsvej 100, Aarhus N DK-8200, Denmark; 2 Department of Cardiology, Aarhus University Hospital Skejby, Brendstrupsgaardsvej 100, Aarhus N DK-8200, Denmark; 3 MR-center, Aarhus University Hospital Skejby, Brendstrupsgaardsvej 100, Aarhus N DK-8200, Denmark; 4 Department of Surgery, Beth Israel Deaconess Medical Center, Harvard Medical School, 330 Brookline Avenue, Boston, MA 02215, USA

## Abstract

**Background:**

Intramyocardialhemorrhage (IMH) reflects severe reperfusion injury in acute myocardial infarction. Non-invasive detection of IMH by cardiovascular magnetic resonance (CMR) may serve as a surrogate marker to evaluate the effect of preventive measures to reduce reperfusion injury and hence provide additional prognostic information. We sought to investigate whether IMH could be detected by CMR exploiting the T1 shortening effect of methemoglobin in an experimental model of acute myocardial infarction. The results were compared to T2-weighthed short tau inversion recovery (T2-STIR), and T2*-weighted(T2*W) sequences.

**Methods and results:**

IMH was induced in ten 40 kg pigs by 50-min balloon occlusion of the mid LAD followed by reperfusion. Between 4–9 days (average 4.8) post-injury, the left ventricular myocardium was assessed by T1-weigthed Inversion Recovery(T1W-IR), T2-STIR, and T2*Wsequences. All CMR images were matched to histopathology and compared with the area of IMH. The difference between the size of the IMH area detected on T1W-IR images and pathology was −1.6 ± 11.3% (limits of agreement, -24%–21%), for the T2*W images the difference was −0.1 ± 18.3% (limitsof agreement, -36.8%–36.6%), and for T2-STIR the difference was 8.0 ± 15.5% (limits of agreement, -23%–39%). By T1W IR the diagnostic sensitivity of IMH was 90% and specificity 70%, for T2*W imaging the sensitivity was 70% and specificity 50%, and for T2-STIR sensitivity for imaging IMH was 50% and specificity 60%.

**Conclusion:**

T1-weigthednon-contrast enhanced CMR detects IMH with high sensitivity and specificity and may become a diagnostic tool for detection of IMH in patients with myocardial infarction.

## Background

A marked decline in mortality from acute myocardial infarction (AMI)has been achieved over the last decade. Primary percutaneous coronary intervention and thrombolysis has contributed to the improved prognosis by reducing the amount of myocardial necrosis because blood flow to the jeopardized myocardium is rapidly restored [1,2]. However, prolonged coronary occlusion followed by reperfusion may cause microvascular injury and prompt the formation of intramyocardial hemorrhage (IMH) [3-5]. Recently, it has been shown that the presence of IMH is associated with adverse left ventricular remodeling and poor prognosis [6-8]. A non-invasive imaging modality capable of identifying IMH may provide further prognostic information and evaluate the efficacy of intervention directed towards reducing reperfusion injury in patients with AMI.

Cardiovascular magnetic resonance (CMR) provides a comprehensive evaluation of myocardial function and morphology in patients with AMI including myocardial viability, perfusion, infarct size, microvascular obstruction (MVO), and edema [9-11]. Previous studies have demonstrated the potential of CMR for assessing IMH as a hypo intense signal intensity or “negative contrast” by exploiting the T2 and T2* shortening effect caused by the elevated myocardial densities of paramagnetic hemoglobin degradation products (deoxyhemoglobin, methemoglobin) or blood degradation products (ferritin and hemosiderin) [7,8,12,13]. However, the separation of the hypo intense signal intensity associated with IMH is challenging because normal myocardium also appears dark on T2-STIR and T2*W images. Furthermore, hypo intense signals attributable to other causes, e.g. tissue interface or motion artifacts may also be difficult to distinguish from IMH. To facilitate the detection of IMH, a CMR approach that detects IHM with hyper intense signal intensity or “positive contrast” would be advantageous. We hypothesized that IMH could be reliable detected using a T1W IR sequence that exploits the short T1 relaxation time of methemoglobin resulting in hyper intense signal intensity [14-16]. In this study, we aimed to validate this approach in a porcine model expressing IMH [17]. We also aimed to compare these results with those of the T2-weighthed short tau inversion recovery (T2-STIR), and T2*-weighted (T2*W) sequences currently used for CMR assessment of IMH [7,8,13].

## Methods

### Animal model

Ten female Danish Land Race pigs weighing 40 kg were used for the experiments. All the pigs were treated in accordance with the Danish law on animal experiments.

The pigs were pre-sedated with an intramuscular injection of stressnill (1 ml/kg), and midazolam (1 ml/kg). After induction of anesthesia with intravenous propofol (5 mg/kg) and endotracheal intubation, anesthesia was maintained with isoflurane (2.5%) in oxygen and continuous rate infusion of fentanyl (3 mg/kg/hr). The pigs were mechanically ventilated with a tidal volume of 450 ml (respiratory rate 12/min).

The right common femoral artery was exposed by a surgical cut down, and a 8 F introducer sheath was inserted into the artery followed by a bolus injection of heparin (100 IU/kg) administrated through the sheath. Coronary occlusion was induced by placing a 2.5 mm over-the-wire angioplasty balloon in the LAD distal to second diagonal branch artery and inflating it to 5 atm. The LAD was occluded for 50 minutes, shown to be sufficient for creating IMH in a previous pilot study. Subsequently the balloon was deflated and removed. An angiogram was performed after balloon inflation and deflation to confirm coronary occlusion and coronary reperfusion, respectively. To prevent ventricular fibrillation, 150 mg of amiodarone was administered through the sheath prior to the induction of myocardial infarction. If ventricular fibrillation was encountered, non-synchronized direct current defibrillation (200 J) was performed. With ultrasound gel applied, the paddles were pressed against the anterior chest wall above the sternum on the right side and below the sternum on the left side.

### Cardiac magnetic resonance

All pigs underwent CMR between three and nine days (average 4.8 days, range 3–9 days) after the ischemia-reperfusion injury, using the same sedation and respiratory protocol as described above. The anesthetic regimen yielded a low cardiac frequency of around 40 beats/min. CMR was performed on a 1.5 T MR system (Intera, Philips Medical Systems, Best, The Netherlands) using a five-element cardiac synergy coil. All pigs were imaged in the supine position. After a survey scan to localize the heart and diaphragm, a multi-heart phase steady-state free precession (BTFE) cine scan (repetition time (TR) 2.6 ms; echo time (TE) 1.3 ms; flip angle 60°; 50 heart phases) was obtained in the cardiac short-axis, vertical and horizontal long axis. In the cardiac short-axis, the LV was completely encompassed by contiguous 8 mm slices. To accomplish optimal delineation of left ventricular (LV) myocardium, 10–12 short-axis images was acquired encompassing the entire left ventricle using balanced steady state free precession (SSFP) scans with the following parameters: Echo time (TE) = 1.3 ms; repetition time (TR) = 2.6 ms; number of averages (NSA) = 1; slice thickness (ST) = 8 mm; in-plane image resolution = 2 x 2 mm.

T1W IR (GRE), T2-STIR (SE), and T2*W (GRE) imaging was obtained in the same short-axis slices. All three sequences were navigator-gated, free-breathing and cardiac-triggered. The specific imaging parameters for each sequence are summarized in Table 1. Before the acquisition of the T1W IR sequence a TI scout (Look Locker sequence) was performed for the purpose of obtaining the most appropriated TI to null the signal intensity from blood. Typically, the TI was found to be optimal at approximately 500 ms.

**Table 1:** Parameters

**Technique**	**T1W IR**	**T2-STIR**	T2*W
	**3D Black-blood**	**2D Black-blood**	**2D Black-blood**
	**GRE**	**FSE**	**GRE**
TE, ms	1.6	100	14
TR, ms	5.0	2000	15
Echo train length	15	20	48
Matrix	248 x 248	248 x 248	248 x 248
Coverage, slices	12	12	12
Slice thickness, mm	8	8	8
Interslice spacing, mm	0	0	0
In-plane resolution, mm	1.2 x 1.2	1.2 x 1.2	1.2 x 1.2
Scan time, min	2.48	4.00	3.24

*T2-STIR* = Short tau inversion recovery, *T2W* = T2-weighted, *T1W IR* = T1-weighted inversion recovery, *FSE* = fast spin echo, GRE = gradient echo, TR = repetition time, TE = echo time.

Following this, gadolinium enhanced first-pass myocardial perfusion and late gadolinium enhancement (LGE) was performed for the purpose of identifying areas of MVO. An intravenous bolus dose of 0.2 mmol/kg Gd-DTPA (Gadobutrol, Gadovist, Bayer Schering Pharma, Berlin) was administered manually. First-pass perfusion imaging was performed using a fast gradient echo sequence with the following parameters: Repetition time (TR) 2.3 ms, echo time (TE) 1.3 ms, flip angle 18°, spatial resolution (2.8) mm x (x3.0) mm x 10 mm, field of view (FOV) range 360 mm, 3 slices acquired in the left ventricular (LV) short-axis using a 10 mm inters lice gap.

Fifteen minutes after gadolinium injection, a 'Look Locker' sequence was performed to obtain the most appropriate TI to null the signal intensity of normal myocardium. The TI was in the range of 300-350 ms. Subsequently, LGE was acquired using a 3D phase sensitive inversion recovery-prepared T1-weighted gradient echo sequence with the following parameters: TR 4.9 ms, TE 1.9 ms, flip angle 15°, spatial resolution 1.35 mm x1.35 mm x10 mm, FOV 350 mm, 8 slices was acquired in the left ventricular (LV) short-axis and no inters lice gap.

### Pathology

After completion of CMR imaging, the pig was kept under anesthesia and transported directly to the operating room. A midline sternotomy was performed, and a snare was placed around the LAD distal to the second diagonal branch, at the same level as the previously performed balloon occlusion. The heart was perfusion stained by an injection of 25 ml 10% Evans blue dye into the left atrial appendage to delineate the area of risk (AAR). Subsequently, the animal was euthanized and the heart was excised. The heart was then cut into 5–7 consecutive 8 mm-thick slices in short-axis planes in concordance with the previously recorded CMR images. Each slice was photographed with a digital camera (Nikon, Tokyo, Japan) for the purpose of registering IMH within the AAR.

## Data analysis

### CMR images

The CMR images were analyzed by two observers using the research software Segment [18]. First, to define the myocardial volume of the left ventricle, the endocardial and epicardial borders of the left ventricle were manually delineated in each of the short-axis SSFP images. The myocardial contours were then copied to the corresponding T1W IR, T2-STIR, and T2*W images. The presence and extent of myocardial hemorrhage was assessed using the following semi-automatic approach. A Region of Interest (ROI) was placed in a homogenous region of the normal myocardium and the relative mean signal intensity (SI) was measured. On T1W IR images, myocardium with a mean signal intensity more than 2 SD above the mean ROI SI was defined as IMH, on T2*W images myocardium with SI 2 SD below the mean ROI SI was defined as IMH, and on T2-STIR images, areas of hypo intense signal intensity within the core of the AAR, i.e., myocardium with a mean signal intensity below 2 SD of that of the periphery of the AAR, were considered to represent IMH [7]. The IMH size in each short-axis was expressed as a percentage of the myocardial surface area (IMHarea/myocardium area x 100%).

Contrast to noise ratio (CNR) was calculated for T1W IR, T2-STIR, and T2*W images from the signal to noise ratios (SNR) of IMH and healthy myocardium:

(1) $$\text{CNR}={\text{SNR}}_{\text{IMH}}–{\text{SNR}}_{\text{healthy myocardium}},\left(\text{SNR}={\text{SI}}_{\text{mean}}/{\text{SD}}_{\text{noise}}\right)$$

CMR mapping was performed on the short-axis images using the MOLLI sequence [19]. T2 values were obtained by a standard multi echo protocol on the scanner. T1 and T2 values were estimated using exponential fitting with in-house developed MATLAB 7.10 code (The Math Works Inc., Natick, MA, USA).

Two examiners blinded to the pathology data visually inspected the CMR images and scored them as being either positive or negative for IMH. On T1W IR images, IMH was defined as an area with hyper intense signal intensity, on T2*W images IMH was defined as a hypo intense area that was clearly distinguishable from the surrounding myocardium, and on T2-STIR images it was defined as a hypo intense area in the core of a hyper intense area [7].

### Pathology

One observer (SAT) reviewed all the digital photos of the freshly cut myocardial slices. Based on the presence or absence of a distinct red blood-stained area in the left ventricular myocardium, each segment was being categorized as being either positive or negative for IMH. The IMH size was manually measured using the Adobe Photoshop software (Adobe Systems Inc., San Jose, CA, USA). The IMH size in each short-axis slice was expressed as a percentage of the myocardial surface area (IMH area/myocardium area x 100%).

### CMR compared with histopathology

The short-axis CMR images of the myocardium were matched with the corresponding photos of the histopathology by using the distance from the apex and gross morphological features such as the overall size and shape of the myocardium.

The CMR findings of each of the T1W IR, T2-STIR and T2*W images were compared to the pathological findings and the sensitivity and specificity to IMH was determined for each sequence. Finally, the area of hyper intense signal intensity on the T1W IR and the hypo intense signal intensity on T2-STIR and T2*W images were compared to the IMH area detected by the pathology.

### Statistical analysis

Bland-Altman analysis was used to compare agreement between the CMR methods and pathology [20]. The underlying assumption of slice independence for each pig was assessed using one-way ANOVA such that the quantitative comparison between CMR and pathology was done for each left ventricular short-axis slice. Interobserver reproducibility was assessed with a 1-way random, single-measure intraclass correlation coefficient (ICC) using SPSS software version 18.0 (SPSS Inc. Chicago, IL).

## Results

Ten pigs were subjected to the experimental protocol; two of the pigs died while awaiting CMR investigations leaving eight pigs for further investigation. All CMR examinations and pathological procedures were successfully performed.

### Pathology

The pathology showed that all the pigs had suffered myocardial infarction and IMH was observed in six of the eight pigs with an average area of 19% CI_95_ [14% to 23%] of the LV. The two pigs that did not show IMH on pathology were excluded from the Bland-Altman analysis since this statistical analysis aimed to evaluate the agreement for quantification of the area of IMH. The IMH sensitivity and specificity for the quantitative analysis of T1W IR, T2*W, and T2-STIR images are summarized in Table 2 for each pig and per short-axis slice.

**Table 2:** Sensitivity and specificity values for detection of IMH per pig and per short-axis slice using T1W IR, T2-STIR, and T2*W sequences

	**T1W IR**	**T2-STIR**	T2*W
Sensitivity (%) per slice	90	70	50
Specificity (%) per slice	70	50	60
Sensitivity (%) per pig	100	100	83
Specificity (%) per pig	100	0	50

### CMR

Cine images demonstrated impaired regional left ventricular contractility in all pigs corresponding to the LAD infarction with an average ejection fraction of 43% CI_95_ [36% to 51%]. The first pass perfusion scan and LGE showed that MVO was present in all of the eight pigs with IMH. All pigs showed LGE enhancement with an average area of 32% CI_95_ [26% to 39%].

### T1W IR images compared to pathology

**Figure 1:**
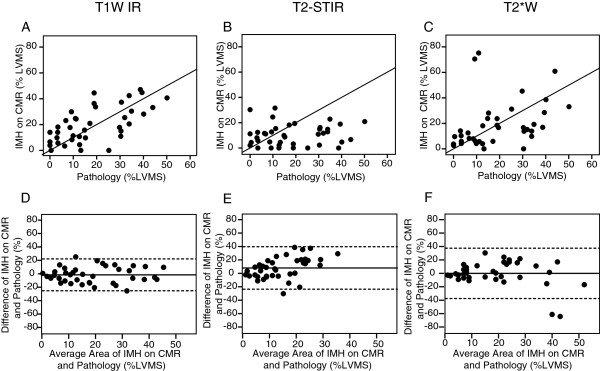
**Scatter plot (A) and Bland–Altman plot (D) for IMH measurements determined by T1W IR and pathology.** Scatter plot (**B**) and Bland–Altman plot (**E**) for IMH measurements determined by T2-STIR and pathology. Scatter plot (**C**) and Bland-Altman plot (**F**) for IMH measurements determined by T2*W and pathology. In the Bland–Altman plots, solid lines represent the mean and dashed lines represent the upper and lower limits of agreement. LVMS = Left Ventricular Myocardial Slice.

**Figure 2:**
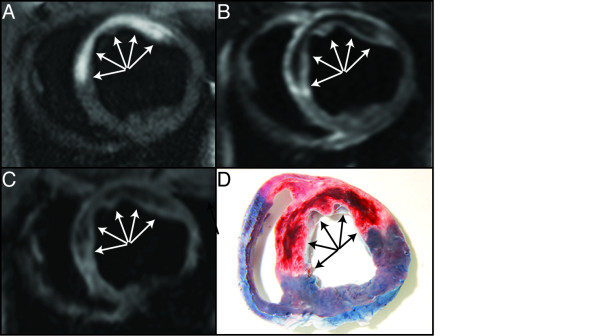
**Short-axis CMR images and corresponding pathology obtained four days following ischemic reperfusion injury.** In the antero-septal myocardium,a distinct hyper intense core region (arrows) is present on the T1W IR image (**A**) while a hypointense core region (arrows) is seen on the T2-STIR (**B**) and T2*W images (**C**). Each of the observed regions corresponds to intramyocardial hemorrhage (arrows) as confirmed by pathology (**D**).

T1W IR images showed hyper intense signal intensity in all six IMH pigs with an average area of 20% CI_95_ [16% to 25%] of the LV (Movie 1) whereas hyper intense signal intensity was not present in any of the IMH negative pigs. The mean difference between the area of IMH detected by pathology comparedwith T1W IR images was: -1.6%, (limits of agreement were −24% to 21%) (Figures 1 and 2).

### T2*W images compared to pathology

**Figure 3:**
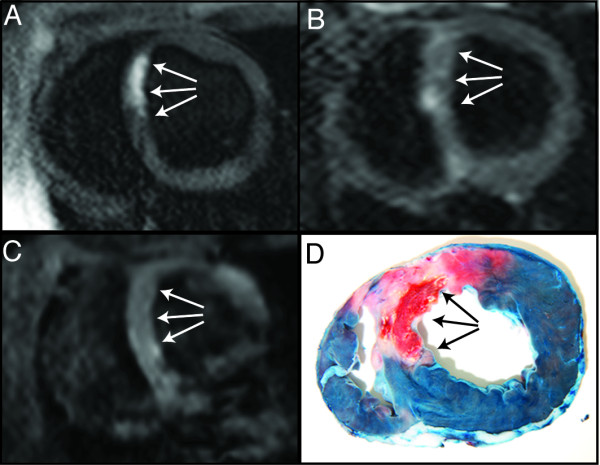
**Short-axis CMR images and corresponding pathology obtained nine days following ischemic reperfusion injury.** In the antero-septal myocardium a distinct hyper intense core region (arrows) is present on the T1W IR image (**A**), whereas only a weak hypointense region (arrows) can be detected on the T2-STIR (**B**) or T2*W images (**C**). The pathology confirms that intramyocardial hemorrhage (arrows) is present in the antero-septal myocardium.

T2*W images showed a hypointense signal intensity in five of the six IMH positive pigs with an average area of 18% CI_95_ [12% to 25%] of the LV and in one of the two IMH negative pigs. The mean difference between the area of IMH by pathology compared with T2*W images was: -1.0% (limits of agreement were −23% to 39%) (Figures 1 and 2).In one of the pigs, IMH could not be detected on T2*W images and only weakly on T2-STIR images despite verification of severe IMH by pathology (Figure 3).

### T2-STIR images compared to pathology

**Figure 4:**
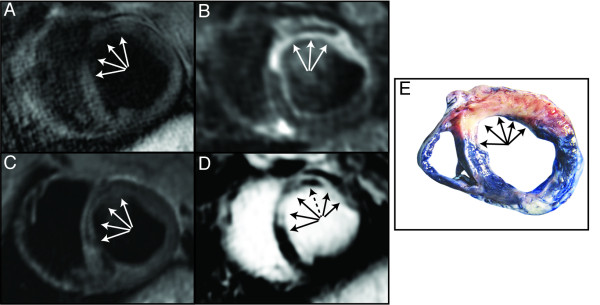
**Short-axis CMR images and corresponding pathology obtained five days following ischemic reperfusion injury.** No hyper intense region is present on the T1W IR image (**A**), whereas a hypo intense core region (arrows) can be detected on the T2-STIR (**B**) and T2*W images (**C**). The LGE image (**D**) reveals substantial scar formation corresponding to the hypo intense T2-STIR/T2*W areas. Inside the LGE hyper intense area, a dark region is present (dashed arrow) indicative of MVO. The pathology image (**E**) confirms that no intramyocardial hemorrhage (arrows) is present in the antero-septal myocardium.

On T2-STIR images a hypointense signal intensity in the center of a hyper intense region was visually detected in all IMH positive (n = 6) and all IMH negative pigs (n = 2) (Figure 4) with an average area of 10% CI_95_ [8% to 13%] of the LV. The mean difference between the area of IMH by pathology and by T2-STIR images was: 8.0% (limits of agreement were-23% to 39%) (Figures 1 and 2).

### Reproducibility for the assessment of IMH on CMR images

The interobserver agreement for the assessment of IMH on cross sectional T1-weighted CMR images was, 0.95 (p < 0.001,CI_95_ = [0.82 to 0.99]) for T2*-weighted images it was 0.91 (p < 0.001, CI_95_ = [0.670 to 0.98]) and for T2-STIR-weighted images it was 0.90 (p < 0.001, CI_95_ = [0.670 to 0.98]). By visual inspection, rating each image slice as positive or negative for IMH, no disagreement was present between the two observers.

### CNR comparisons

The CNR of T1W (105.7, CI_95_ [−80.3 to 291.7]) was superior to T2-STIR (13.6, CI_95_ [−10.7 to 37.9])and T2*W imaging (20.0, CI_95_ [−22.3 to 62.3]).

### CMR mapping

The T1 values of areas containing IMH were shorter (569 ms, CI_95_ [377 to 761]) than in the remote myocardium (707 ms, CI_95_ [587 to 827]). Likewise, T2 values of IMH were shorter (38 ms, CI_95_ [22 to 54]) than in the remote myocardium (54 ms, CI_95_ [36 to 72]).

## Discussion

The results of this study show that CMR using T1W IR accurately distinguishes between the presence and absence of IMH in porcine myocardium exposed to ischemia-reperfusion injury. The area of hyper intense signal intensity using T1W IR was in agreement with IMH as verified and quantified by pathology. The study also demonstrated that, the T1W IR imaging yielded a higher sensitivity and specificity for IMH as well as a superior agreement with pathology compared toT2-STIR and T2*W imaging that in previously studies have been used for IMH imaging [7,8,13]. Further, acquisition times using T1W IR are inherently shorter than T2-STIR and T2*W yielding added benefits in a clinical setting with higher cardiac frequencies and correspondingly shortened diastolic rest periods suitable for cardiac imaging.

These data constitute the first evidence that CMR T1W IR imaging allows IMH detection by demonstrating hyper intense signal intensity in the left ventricular myocardium.

The observed ability of CMR T1W IR imaging to detect IMH by exploiting the T1 shortening effect of met hemoglobin is consistent with previous studies demonstrating that this technique is highly sensitive in detecting hemorrhage within the brain and in carotid atherosclerotic plaques [14-16].

In this study, T1W CMR demonstrated improved accuracy compared to T2-STIR and T2*W techniques for the detection of IMH when the CMR examination was performed subacute. The reason for this is most likely that the T1-weighted technique depicts IMH (methemoglobin) by means of hyper intense signal intensity and normal myocardium with hypointense signal intensity. In contrast, the T2-STIR and T2*W techniques depict both IMH and normal myocardium by means of hypointense signal intensity. The T1-weighted technique may therefore provide superior image contrast and thereby allow for improved IMH identification.

We found that in one of the pigs, IMH could not be detected onT2*W images and only weakly on T2-STIR images despite verification of severe IMH by pathology (Figure 3). In this animal, CMR imaging was performed 9 days after the ischemia-reperfusion injury, thus the delay time for this pig was longer when compared to the remaining pigs (average 4.8 days). At this time stage extracellular formation of met hemoglobin may be present, which has neither a T2 shortening effect nor a particularly strong susceptibility effect [15,21]. Consequently, hypo intense signal intensity would not be generated on T2*W or T2-STIR images at this time stage. This implies that CMR performed sub acutely is suboptimal when using the T2-STIR and T2*W techniques. Further investigation is needed to confirm the presence and range of such a time window for detection of IMH by T2-STIR and T2*W techniques.

On the T2-STIR images, we observed the opposite situation: In two of the pigs, a hypo intense infarct core was detected in the myocardium, but IMH could not be detected in the corresponding pathology (Figure 4). A hypo intense infarct core on T2-STIR images should therefore not be considered specific for IMH. Indeed, it may also represent other pathologies such as MVO or non-edematous scar tissue. Therefore, the combination of T1W andT2-STIR CMR may differentiate between MVO with and without IMH in reperfused AMI. T1W CMR provides a new diagnostic tool for the detection and quantification of IMH in reperfused myocardial infarction that may predict adverse cardiac events in patients with AMI. This technique may also be a useful instrument to evaluate treatment response to new adjunctive therapy aimed at reducing reperfusion injury.

### Limitations

While the data were convincing in demonstrating the ability of T1W IR imaging for detection of IMH, the sample size was small. Larger studies would be useful to confirm our findings. Matching CMR images to pathology is a challenging task that involves some inaccuracy, even when matching is carefully performed. Since there was no disparity in slice thickness between histopathology slices and CMR images (both 8 mm), we believe that this inaccuracy was minimal. In the present study, the ability of T1W IR, T2-STIR, and T2*W techniques to detect IMH were assessed in the subacute phase after ischemia-reperfusion. The diagnostic performance of the different CMR techniques in the acute and chronic phase when the hemoglobin breakdown products is mainly based on the form of deoxyhemoglobin and hemosiderin, respectively, is likely to be different and needs further investigation.

## Conclusion

T1W IR CMR enabled detection of porcine IMH with a high sensitivity and specificity and has the potential to become a useful non-invasive diagnostic tool for assessment of IMH in patients with myocardial infarction.

## Competing interests

The authors declare that they have no competing interest.

## Authors’ contributions

SFP formulated the study, carried out all the procedures related to the animal model, contributed to performing the CMR scans, contributed to the statistical analyses, obtained all illustrations, wrote the manuscript, and merged all feedback from the co-authors into the final manuscript. ST performed the analysis of the acquired CMR and histological data, contributed to the statistical analyses, the illustrations and drafting the manuscript and revised it critically for intellectual content. MPR contributed to the study design, drafting the manuscript, and revised it critically, for intellectual content. WP took part in formulating the study, revised the manuscript critically and contributed to important intellectual content of the manuscript. SR contributed to the CMR sequence setup, revised the manuscript critically and contributed to important intellectual content of the manuscript. HEB revised the manuscript critically and contributed to important intellectual content of the manuscript. ESSH revised the manuscript critically and contributed to important intellectual content of the manuscript. WYK contributed to the study design, the CMR sequence setup, drafting the manuscript and revised it critically for intellectual content. All authors have read and approved the final manuscript.
